# The effect of nicotine on threat avoidance behaviour in healthy non-smokers

**DOI:** 10.1007/s00213-025-06789-9

**Published:** 2025-04-22

**Authors:** Madeleine Mueller, Christoph Korn, Jan Haaker

**Affiliations:** 1 Department of Systems Neuroscience, University Medical Center Hamburg-Eppendorf, Martinistr.52, 20251 Hamburg, Germany; 2 Department of General Psychology and Cognitive Neuroscience, Charlotte Fresenius University, Alte Rabenstraße 32, 20148 Hamburg, Germany; 3 Section Social Neuroscience, Department of General Psychiatry, University of Heidelberg, Heidelberg, Germany

**Keywords:** Aversive learning, Avoidance, Nicotine

## Abstract

**Rationale:**

Developing adaptive strategies for survival relies on distinguishing danger from safety through aversive learning mechanisms. Chronic and acute nicotine exposure have been linked to impaired aversive learning and reduced discrimination between threat and safety. Yet, it is unclear if nicotine also impacts one behavioural consequence of aversive learning, which is the avoidance of threats.

**Objectives:**

This preregistered study examines how acute nicotine influences costly avoidance behaviour in non-smokers.

**Methods:**

To this end, healthy non-smoking participants (*n* = 66) received either 1 mg nicotine or a placebo in a double-blind design and underwent an active avoidance task. During acquisition, participants could choose between a safer but longer path to reach their goal or a shorter path (less effort) with a higher chance of receiving an aversive outcome in the form of an electrical stimulus. During uninstructed extinction, both paths no longer contained the risk of an aversive outcome and participants could learn this new safety association by trial and error. Finally, an instructed extinction phase indicated complete safety.

**Results:**

Contrary to our pre-registered hypotheses, participants with nicotine intake showed a trendwise reduced avoidance of aversive outcomes, compared to placebo controls. Further analysis revealed however that nicotine did not enhance safety learning during extinction in the nicotine group, as compared to controls.

**Conclusions:**

In conclusion, this study strengthens the evidence that nicotine alters learning to identify threat and safety, which is also transferred to avoidance behaviour.

**Supplementary Information:**

The online version contains supplementary material available at 10.1007/s00213-025-06789-9.

## Introduction

Developing adaptive strategies to avoid danger while being certain of what is safe is fundamental for survival. Aversive learning is an important mechanism to distinguish between danger and safety. It further enables to adjust the behaviour accordingly and balance between the avoidance of threats and efforts to be safe.

Nicotine has been found to impair adaptive aversive learning and memory (Kutlu et al. 2018; Mueller et al. 2024b). Specifically, acute nicotine administration dose-dependently impairs the discrimination between threatening and safe contexts in rodents (Kutlu et al. 2014). Similarly, reduced discrimination between threatening and safe stimuli was also found in human non-smokers receiving nicotine compared with placebo controls (Mueller et al. 2024b). Although differential learning was impaired by nicotine (decreased differential activation of the hippocampus and amygdala), nicotine administration also led to increased physical arousal, measured as increased skin conductance responses. In line with these findings are studies in smokers, showing less discrimination between threatening and safe (Haaker et al. 2017) and exhibiting heightened fear responses to stimuli previously learned as safe, in contrast to non-smokers (Mueller et al. 2022). Since previous studies on nicotine on threat learning used (passive) classical conditioning, it is unclear how nicotine alters avoidance of threat—the behavioural answer to identified threats and safety. It is unknown if the effect of nicotine on aversive learning also impacts decision making and transfers to behaviour.

Decision-making models suggest that individuals weigh each available option to determine its personal value and then choose the option they find most valuable (Levy and Schiller 2020). Making such decision in the face of threats often confronts individuals with the dilemma of costly avoidance (van Meurs et al. 2014; Pittig and Scherbaum 2020; Wong and Pittig 2022): One option is more dangerous than the other, but less costly (e.g., provides more rewards or requires less effort). This option is compared to a more costly but safer option (that allows to avoid the danger). Making such decision demands the participants to weight the higher threat and lower cost of one option against the lower threat and higher cost of another option. In other words, the participants face the decision to avoid a higher threat by the increased cost of the lower threat (or safe) option.

**Fig. 1:**
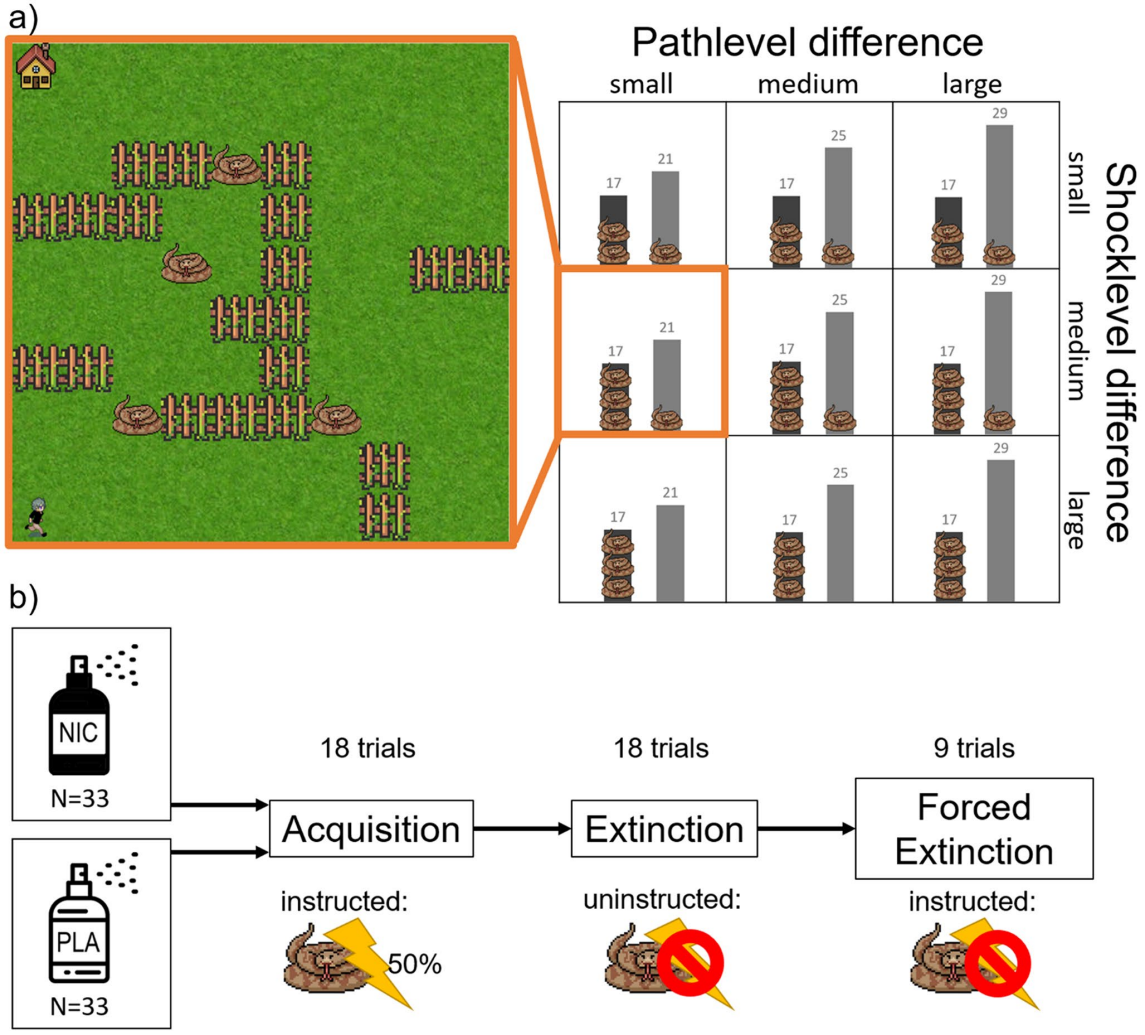
**a**) Paradigm. Participants could move using the arrow keys on the keyboard and were instructed to walk home, indicated by the house. They could choose between two paths, where the shorter path contained more snakes (shock probability per snake 50%). Three level differences of path and shock were implemented into the paradigm. **b**) Procedure. Participants were randomly and double-blinded sorted into either group and accordingly received either 1 mg nicotine or a placebo. The experiment consisted of three phases. During acquisition, participants were instructed that snakes might predict an aversive electric stimulus that was adjusted prior to the experiment to be unpleasant. After 18 trials acquisition, the uninstructed extinction phase started (also 18 trials), where snakes were not predictive for an aversive outcome. Finally during forced extinction, participants were instructed that snakes were no longer predictive for an aversive outcome

In our paradigm, we provide individuals with such a costly avoidance paradigm that includes two available options: A dangerous path, with a higher probability for aversive outcomes (unpleasant electric stimuli) and a safer path, with a lower probability for aversive outcomes. The safer path is associated with an increased effort, since it is longer than the more dangerous path and results in more button presses to reach the goal. Here, costly threat avoidance is therefore implemented as choosing the more effortful, but safer path. To implement parametric differences in the balance between effort and threat avoidance, we included combinations of several differences in effort between the safe and dangerous pathlength and differences in their aversive outcomes (Fig. 1a). The different levels of path length and aversive outcome allow us to study subtle differences in behaviour and further avoid ceiling effects. Furthermore, we examined participants’ costly avoidance behaviour as a function of different threat contexts in the experiment, realised in phases of aversive outcome acquisition, uninstructed extinction of the aversive outcomes, followed by an instructed extinction phase. These phases of our experimental paradigm are based on a well-established standard in the fear conditioning community, using fear acquisition and extinction training in the laboratory (Lonsdorf et al. 2017).

We examined the impact of nicotine on avoidance behaviour in healthy non-smokers and, as preregistered (https://osf.io/evq72), we expected that the nicotine group will exhibit increased avoidance behaviour, indicated by a higher effort to avoid more aversive outcomes (in the dangerous path), compared to the placebo group during all three phases of the experiment. Nicotine has been shown to impair aversive learning and memory, disrupting the ability to discriminate effectively between safe and dangerous situations, both in rodent experiments, as well as our own experiments in humans (Kutlu et al. 2014, 2018; Mueller et al. 2022, 2024b). Impaired learning can make it harder for individuals to assess a situation’s potential dangers, which may increase their tendency to avoid risks and choose safer options. Based on this, we expected the nicotine group to be more likely to choose the safer option and exhibit increased avoidance compared to the placebo group.

## Methods

### Participants

For this study, 66 healthy, non-smoking participants between ages of 18 and 40 were included in the final analysis as pre-registered (Table 1; see preregistration for power analysis: https://osf.io/evq72; open science framework: preregister number/clinical trial code: EVQ72). Individuals confirmed to have no neuropsychiatric disorders, to consume less than 15 units of alcohol per week and no illicit drugs. Non-smokers were defined as not being active smokers at the time of data collection and having smoked fewer than 200 cigarettes in their lifetime. All participants gave written, informed consent to participate and received 40€ reimbursement. The study was approved by the local ethics committee (Ethikkommission der Ärztekammer Hamburg, PV5514) and complies with the Declaration of Helsinki.

**Table 1:** Demographics per group. Group statistics were calculated using an independent samples t-test with each demographic score as the dependent variable and group as the grouping variable (marked as^1^) or using a Chi-Square test of independence with contingency tables for binomial data (marked as^2^)

	Nicotine group	Placebo group	Group statistics
Sample size	33	33	
gender	70.97% female	51.61% female	*p* = 0.12^2^
Age	25.84 (4.41)	26.42 (4.59)	*p* = 0.613^1^
Alcohol consumption [units per week]	1.74 (2.02)	1.18 (1.67)	*p* = 0.236^1^
Coffee consumption [cups per day]	1.0 (1.03)	0.99 (0.84)	*p* = 0.978^1^
Body Mass Index	22.51 (2.48)	24.16 (4.08)	*p* = 0.061^1^
STAI-T (**S**tate/**T**rait **A**nxiety **I**nventory - **T**rait)	31.77 (6.09)	31.24 (6.72)	*p* = 0.742^1^
STAI-S (**S**tate/**T**rait **A**nxiety **I**nventory - **S**tate)	44.48 (2.49)	45.12 (2.56)	*p* = 0.317^1^
Guessed correctly when asked which group they were in	54.54%	36.36%	*p* = 0.138^2^
AO post-experimental unpleasantness rating [0–10]	6.12 (1.14)	6.52 (1.15)	*p* = 0.167^1^

### Task structure

Prior to nicotine administration, participants completed the State-Trait Anxiety Inventory (STAI-S (state)/STAI-T (trait) (Spielberger 1983). Then, participants were randomly and in a double-blinded manner assigned to either the nicotine group, receiving 1 mg of orally administered nicotine spray (Nicorette^®^, Johnson & Johnson GmbH), or the placebo group (Fig. 1b). The dose was determined by pilot studies and has been used in previous studies with robust nicotine effects and few side effects (Mueller et al. 2024b). Peak plasma nicotine concentrations typically begin 10 min after oral nicotine administration (Kraiczi et al. 2011). To ensure that the tasks was performed after peak nicotine levels had been achieved, both groups performed the avoidance task fifteen minutes after nicotine spray administration. In this task subjects are instructed to move a figure on the screen from a start-point into a house using the arrow keys on the keyboard. To get to the house, participants can choose between two paths with different lengths. Both of these paths can contain different amounts of snakes (zero up to three) and if the participant moves their figure across a snake they receive an electrical stimulus (i.e. an aversive outcome) in 50% of the trials (randomised). After oral spray administration, the intensity of the aversive outcome was calibrated for each participant to be unpleasant but not painful.

To examine the relationships between different pathlengths and amounts of aversive outcomes, we created a 3 × 3 condition structure: The participants could choose between a short path (“ideal route” always 17 steps) or a long path (See Figure S1 for an example of a movement pattern with the shortest possible routes). Depending on the trial, the long path has a large difference (= 12 step difference), a medium difference (= 8 step difference) or a small difference (= 4 step difference) compared to the length of the short path. Participants could use the “ideal route”, but as they were free to move along the chosen path, they might need more steps than the minimum to reach the goal. The structure of the conditions further include three different amounts of aversive outcomes at both paths, where the shorter path always contains more snakes (and hence higher probability of aversive outcomes). There are large differences in aversive outcomes between two paths (= 3 aversive outcomes in short path vs. 0 aversive outcomes in long path), medium differences (= 3 aversive outcomes in short path vs. 1 aversive outcome in long path) or small differences (= 2 aversive outcomes in short path vs. 1 aversive outcome in long path)). As mentioned before, the inclusion of three different pathlengths and three possible differences of aversive outcomes results in 9 different conditions (see Fig. 1a), which always demands the participant to take more steps to avoid the path that contains more snakes (i.e., aversive outcomes). The effort came from the clicking itself, but also from the extra time it took to walk a longer distance. Participants needed more time when they chose the longer path compared to the short path (short path– long path: t(2968)=-12.53, *p* < 0.001; mean time short path = 10.02s; mean time long path = 12.24s). Participants were not informed of the predetermined number of trials.

### Procedure

The experiment is divided into three phases (Fig. 1b). In the first phase, acquisition (ACQ), the probability of receiving an electrical stimulus from a snake is 50%. Participants were not told the specific rate of reinforcement, but were told they “could receive an electrical stimulus by touching the snake”. This is followed by an extinction phase (EXT) with a 0% probability of receiving an aversive outcomes, which is not instructed and takes place directly after the ACQ. Finally, a forced extinction (fEXT) follows. Before this last phase, the test subjects are instructed that snakes are not predictive anymore for electrical stimuli. Each of the 9 conditions (see task structure) are each presented twice during ACQ and EXT and once during forced EXT (18 + 18 + 9 = 45 trials). As the forced EXT was instructed to be safe, we expected fast learning rates and therefore only included 9 trials. Trials of each condition are presented randomly per phase.

### Statistical analysis

As preregistered, our primary outcome measures were number of steps per trial and we conducted an exploratory analysis for the path decision (short vs. long path) per trial. Both outcome measures are in some way a representation of effort, but the additional analysis of the path decision is a clearer representation of the choice made and is henceforth referred to as ‘decision-based’, whereas the pure number of steps is referred to as ‘effort-based’. We expected the nicotine group to use more steps because they chose the longer and safer route more often than the placebo group at all phases, indicating greater avoidance behaviour.

For the analysis of the numbers of steps, we used a preregistered linear mixed model in R (lme4 package) and for the path decision analysis, we constructed a similarly structed general linear model in R, assuming a binominal distribution (because of the binary nature of the dependent variable).

As preregistered, the linear mixed model included the number of steps per trial per subject as dependent variable. Additionally, random intercepts for subjects were added. Finally, we included the three levels of aversive outcome levels (AOlevel), three levels of pathlength (Plevel), three phases (levels: ACQ, EXT, fEXT) and two groups (levels: nicotine and placebo) into the model (steps~(1|subject) + AOlevel*Plevel*phase*group).

The exploratory general linear model included the path decision per trial per subject as dependent variable. Additionally, we included the same fixed factors as in the preregistered mixed model, i.e., three levels of aversive outcome levels (AOlevel), three levels of pathlength (Plevel), three phases (levels: ACQ, EXT, fEXT) and two group (levels: nicotine and placebo) into the model (pathdecision ~ AOlevel*Plevel*phase*group). The two main analyses of this study (effort-based and decision-based) are related in that increased effort in terms of more steps taken is, by paradigm definition, related to the decision to take a long route. However, the two outcome measures are not highly correlated, so after analysing the effort-based results, we found that the pre-registered effort analysis did not describe and explain behaviour as a whole, and we added the decision-based model (see supplement for analysis including gender and blinding success).

The follow up ANOVA Type 3 was calculated with the car package. To further test the results, estimated marginal means (EMMs) were computed as post-hoc tests (emmeans package) and Bonferroni-Holm corrected.

Our exploratory analysis further included a binomial test where the binary decision between the short and dangerous path or the longer and safer path per phase was calculated with a test value of 0.5, to ensure that path decisions were not made randomly.

To test for learning effects that might influence the current decision based on earlier experiences in the previous trial, we added a prediction error term to a simplified model. This term is calculated based on the number of snakes crossed in the previous trial and the number of associated aversive outcomes. For each snake that elicited an aversive outcome, the prediction error score increased by 0.5 (50% chance of aversive outcome per snake). Similarly, each snake that did not elicit an aversive outcome decreased the prediction error score by 0.5. Thus, the higher the prediction error score in the current trial, the more snakes elicited an aversive outcome in the previous trial, i.e., the more strongly the snakes were associated with an aversive outcome. It would be expected that these learned associations would be part of the decision making process. We added this prediction error score to the decision model (pathdecision ~ phase*group*predictionerror). Trials in which participants ‘tested’ the snakes by walking over them several times were excluded from the analysis (7 trials in total).

## Results

### Effort-based effects

In contrast to our pre-registered hypothesis, we found no group differences regarding the step analysis, indicating that nicotine administration had no influence on effort-based effects.

**Fig. 2:**
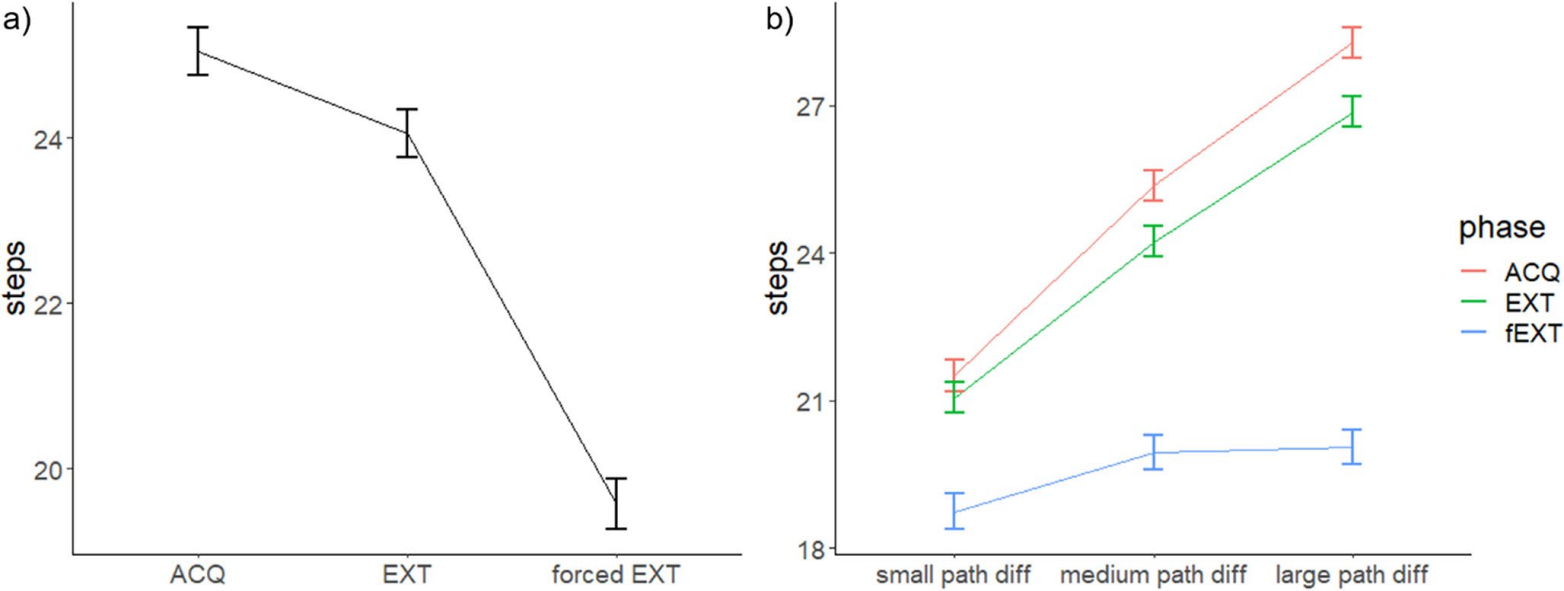
**a**) Effort level over phases. Highest effort for avoidance was found during acquisition. During extinction participants learned that it was not necessary to avoid and decreased their effort. Finally, after being instructed for safety, the effort level to avoid drastically decreased during forced extinction. **b**) Effort level depends on the pathlevel by phase.

However, the effort-based model also aimed to test several effects that confirm paradigm validity, such as an effect of pathlength differences (Plevel), phases of the experiment and differences of aversive outcomes (AOlevel) (see Table 2 and Table S1/S2 for detailed results). The model-based ANOVA supports these effects and revealed a main effect of Plevel. A higher Plevel indicates a higher effort difference between the two paths. We find that number of steps increase from Plevel1 to Plevel3, indicating that even if the effort to avoid increases over Plevels, participants use the safer, higher effort path. The ANOVA further reveals a main effect of phase (Fig. 2a), indicating that participants took the highest amount of steps (highest effort) during the acquisition phase, when compared to the extinction or the forced extinction. During the forced extinction, the effort to avoid was also significantly lower, when compared to the extinction. Hence, instructing the participants that the snake-symbols are no longer followed by an aversive outcome, reduced the need to avoid and to take the longer paths.

Furthermore, the model-based ANOVA indicated an interaction between AOlevel by Plevel, showing that the lowest Plevel has the lowest number of steps for all three AOlevel, while the highest Plevel has the highest number of steps for all three AOlevel (all corrected p-values < 0.001; Table S2). Further and counter-intuitively, participants at the medium Plevel took more steps at the lowest AOlevel compared to the medium and highest AOlevels. However, this is not the case for either the low or high Plevels, where we found no effect of AOlevel dependence on Plevels (all corrected p-values > 0.05; Table S2). The fact that most steps in the medium path level were taken when faced with the lowest aversive outcome level is inconsistent with the expectation of balanced behaviour between threat and cost. Therefore we complement the analysis of the effort (number of steps) with a binary decision model as an analysis of the avoidance decision in the next section to describe participants’ behaviour. The model-based ANOVA also revealed a Plevel by phase interaction (Fig. 2b; Table 2). Looking at each Plevel individually, the number of steps decrease from ACQ to fEXT (all corrected p-values < 0.001, except low Plevel/ACQ– low Plevel/EXT; Table 2/S2). Also, in each phase the number of steps is increasing from low pathlevel to highest pathlevel (all corrected p-values < 0.001, except medium Plevel/fEXT– high Plevel/fEXT; Table 2/S2).

**Table 2:** Effort-based effects. AOlevel = aversive outcome level; Plevel = pathlevel; ACQ = acquisition; EXT = extinction; fEXT = forced extinction. Only effects described in the results section are shown. For a full list of results see supplementary table S1/S2

ANOVA results based on linear mixed model steps~(1|subject) + AOlevel*Plevel*phase*group				post-hoc tests (estimated marginal means), Bonferroni-Holm corrected			
	F	Df	p		t	df	p_corr_
Plevel	50.29	2,2852	< 0.001	Plevel1– Plevel2	-18.17	2852	< 0.001
				Plevel2– Plevel3	-12.5	2852	< 0.001
phase	11.37	2,2852	< 0.001	ACQ– EXT	7.66	2852	< 0.001
				ACQ– fEXT	34.16	2852	< 0.001
				EXT– fEXT	27.91	2852	< 0.001
AOlevel*Plevel	2.74	4,2852	0.027	Plevel2/ AOlevel1– Plevel2/ AOlevel2	3.849	2852	< 0.001
				Plevel2/ AOlevel1– Plevel2/ AOlevel3	3.071	2852	0.018
Plevel*phase	4.26	4,2852	0.002	Plevel1/ACQ– Plevel1/EXT	1.996	2852	0.092
				Plevel2/fEXT–Plevel3/fEXT	-0.379	2852	0.705

**Fig. 3:**
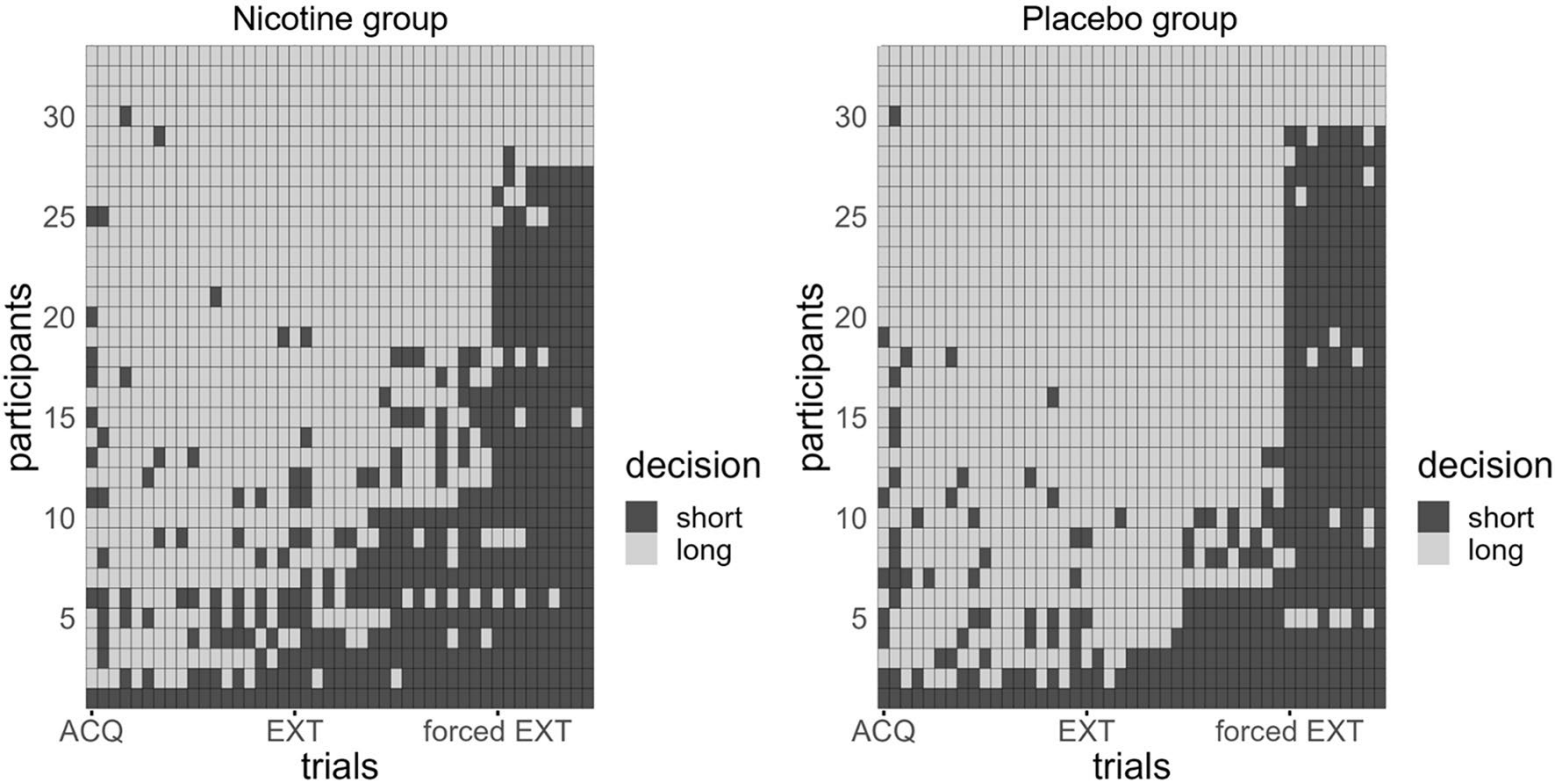
Decisions over time per group. Sorted from least avoidance (always the short path) to most avoidance (always the long path), this figure depicts the decision per group over time. The decreased avoidance during extinction phase is visible in the nicotine group (left)

### Decision-based effects

To follow-up on the difference in the decisions to take the shorter vs. the longer path, a binomial test was used to ensure that path decisions were not at chance level, but depended on the threat context of each phase. In all three phases, there was a difference between the path decisions and the chance level of 0.5, meaning that the decisions between paths were not taken randomly. During the acquisition phase, participants more often chose the longer path (87.5%, *p* < 0.001) than chance. The same effect was found in extinction, showing stronger avoidance by more frequently deciding for the longer path (77.1%, *p* < 0.001), than chance. During forced Extinction, this effect was the other way round. Now that both paths were instructed to be equally safe, more participants decided to use the shorter path, which takes less effort (79%, *p* > 0.001), as compared to chance level.

**Fig. 4:**
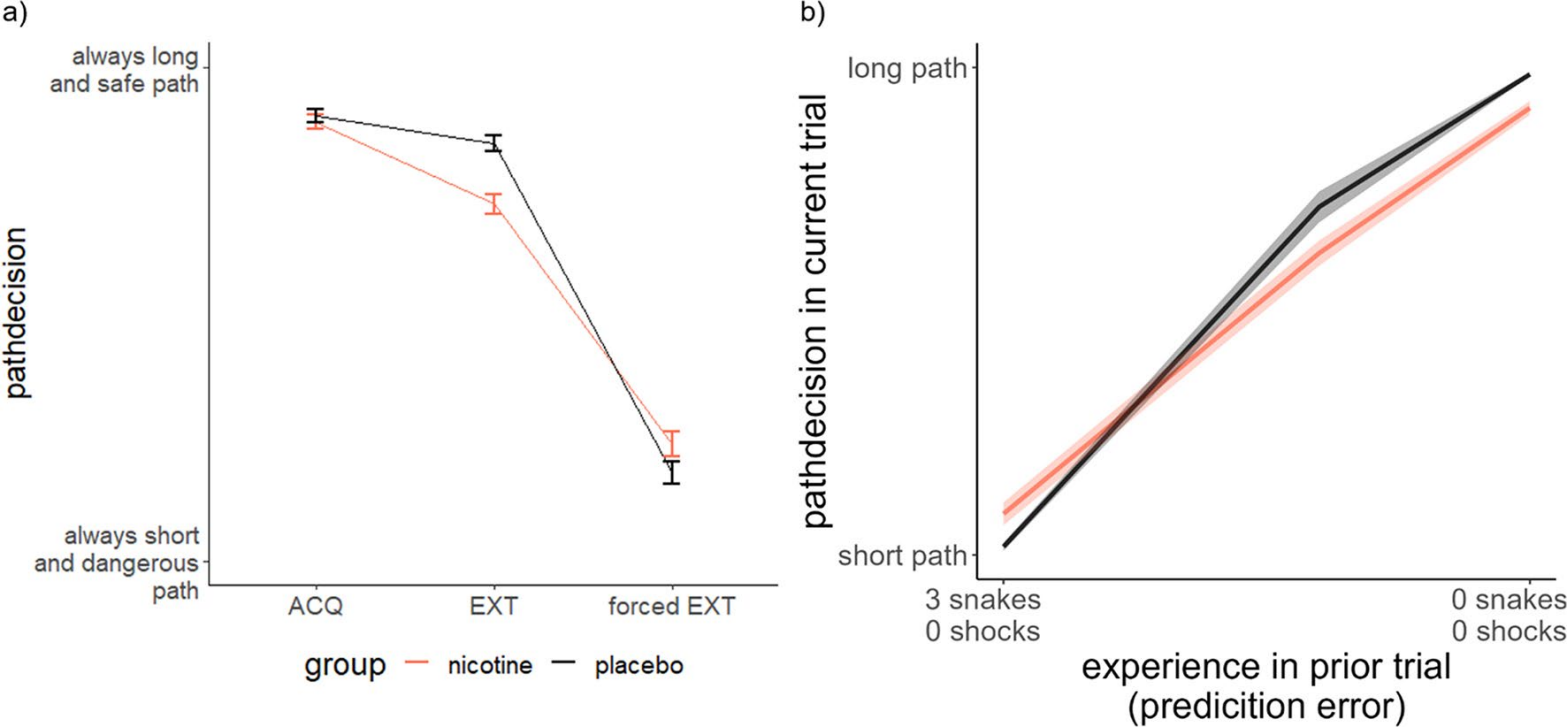
**a**) Path decision. Nicotine administration leads to a decrease in avoidance behaviour during extinction, compared to placebo controls. **b**) Prediction error per group during extinction. The steeper slope in the prediction error in the placebo group compared to the nicotine group indicates that nicotine administration might impair to integrate unexpected omissions of aversive outcomes to learn that previously dangerous situations are now safe. Please note that no aversive outcomes were administered during extinction (i.e., unexpected omissions of aversive outcomes)

The decisions to take the shorter, more dangerous path, as compared to the longer, safer path per group is illustrated in Fig. 3. This figure illustrates that the placebo group might show a stronger preference for the longer path, i.e. avoidance of the dangerous path during extinction. This effect would go against our pre-registered hypothesis. In order to examine group differences in the decision for the avoidance of the shorter, more dangerous path, we constructed a decision-based general linear model and, as before, ran an ANOVA based on this model. The results of this ANOVA revealed a main effect of AOlevel (F(2,2916) = 6.08, *p* = 0.002), indicating that the higher the chance to receive an aversive outcome (higher AOlevel), the more likely participants decided to avoid the shorter and chose the longer, safer path (AOlow– AOmedium: z=-3.76, *p* < 0.001, AOmedium– AOhigh: z=-2.27,*p* = 0.023). A main effect of phase (F(2,2916) = 15.55, *p* < 0.001) mirrored the results from the effort-based analysis, indicating that participants showed the highest avoidance of the short path during the acquisition phase and then this avoidance decreased during extinction, while very little avoidance was observed during forced extinction (ACQ– EXT: z = 6.24,*p* < 0.001, EXT– fEXT: z = 20.75,*p* < 0.001). Furthermore, we found a trend towards a phase by group interaction (F(2,2916) = 2.33, *p* = 0.098; Fig. 4a). Even though, this interaction does not meet our statistical alpha-level, this trend goes against our pre-registered hypothesis that nicotine would increase avoidance of the shorter, more dangerous path. Post-hoc tests revealed a group difference during extinction, in which the nicotine group showed decreased avoidance of the short path, as compared to placebo controls (EXT_nicotine_ - EXT_placebo_: z=-4.791, p_corr_<0.001). We found no influence of nicotine administration on the path decision during acquisition (ACQ_nicotine_ - ACQ_placebo_: z=-0.65, p_corr_=0.516) and forced EXT (fEXT_nicotine_ - fEXT_placebo_: z = 1.73, p_corr_=0.168). In contrast to the effort-based analysis, we did not find an interaction between AOlevel and Plevel (F(4,2916) = 0.29, *p* = 0.884).

### Prediction error analysis

When including the prediction error score into the decision model, a steeper slope of the prediction error indicates that when more aversive outcomes were experienced in the previous trial, the decision in the present trial would be more likely the longer, but safer path.

Further, we found a phase by group by prediction error interaction (F(2,2951) = 7.34, *p* < 0.001), indicating a steeper prediction error slope in the placebo group during EXT, compared to the nicotine group (z=-4.07, *p* < 0.001). This means that the nicotine group decided to take the longer path more often, even when they crossed multiple snakes that did not elicit an aversive outcome in the prior trial, compared to the placebo group. The placebo group was more likely to choose the shorter path, when the previous trial indicated safety (Fig. 4b). Combined with the other results, this means that nicotine generally led to the shorter path being chosen more often (less avoidance), but not because of an enhanced prediction error-based safety learning.

### Side effects

We recorded the intensity of typical side effects, such as a dry mouth, dry skin, vertigo, weariness, blurred vision, nausea or headache on a visual analogue scale of one to seven (1 = no side effect, 7 = extreme side effect). When comparing the rated mean intensity between groups, we found higher intensity of side effects in the nicotine as compared to the placebo group (nicotine– placebo: t(64) = 3.6, *p* < 0.001). When we looked at each side effect separately, we found that not all of the side effects recorded were more severe in the nicotine group. The Mann-Whitney u test showed that only vertigo, weariness and nausea were significantly stronger in the nicotine group (*p* < 0.05).

However, the mean of side effects in both groups was smaller than a rated 2 on the scale (2 = very little side effects; nicotine group = 1.62, sd = 0.79; placebo group = 1.11, sd = 0.22). This indicates that although the nicotine group showed increased side effects, compared to the placebo group, these side effects are overall very weak. At the end of the experiment, participants were asked to guess their group assignment and 45.45% guessed correctly. This is in line with expectations of a successful blinding process, as randomly guessing between the two group options would result in 50% correct guesses.

## Discussion

Our results indicate that acute nicotine in healthy non-smokers might alter decisions to actively avoid threats. We found initial evidence that administration of nicotine reduces the avoidance of potentially dangerous outcomes, especially, when participants learn that these dangers are no longer present. In our previous studies (Mueller et al. 2022, 2024b), we found that nicotine reduced the differentiation between threat and safety (when administered to non-smoker or when comparing smokers to non-smokers). These earlier studies also showed that smokers rated more fear to stimuli that were learned safe and that administration of nicotine to non-smokers elicit higher psychophysiological responses to learned threats and lower neural integration of threat-contingencies in the hippocampus. Hence, our hypothesis was that administration of nicotine to non-smoking participants would increase the avoidance of aversive outcomes. However, our results indicate that nicotine reduces the decisions to take the path that would possibly lead to more aversive outcomes.

Interestingly, this effect would still indicate that administration of nicotine reduces the differentiation between dangerous and safe outcomes and thereby maybe increases the decision of participants to take the dangerous, instead of the more safe option.

We further conducted an analysis of possible safety learning by integrating participants’ experience from the previous trial into the decision model. This model revealed that the lower avoidance in the nicotine group did not rely on better integration of unexpected omissions of aversive outcome during extinction (that would drive safety learning) as compared to subjects that received placebo. This means that enhanced safety learning from the unexpected omission of aversive outcomes was not related to the reduction in avoidance behaviour in the nicotine group, when compared to the placebo controls.

While we found trend-wise evidence that nicotine affected decisions to avoid a threat in our exploratory analysis, we found no difference between the groups in effort (here measured in steps = button presses) within the paradigm. Moreover, nicotine did not affect avoidance of threats in situation that are in fact associated with aversive outcomes (acquisition), but when the aversive outcomes were no longer present (uninstructed extinction phase). Nicotine had no effect on instructed extinction when participants were told they were safe from aversive outcomes.

Based on our unexpected results, different interpretations are possible. It could be argued that nicotine leads to improved adaptive avoidance. Since there are no differences in avoidance between the groups during acquisition, but then during extinction, this could indicate that acute nicotine led to faster learning that the snakes were no longer predictive of an aversive outcome. However, this would be in stark contrast to the literature, which is provides comprehensive evidence that nicotine (acute and chronic) leads to impaired safety learning in humans (Connor et al. 2017; Mueller et al. 2022; Kutlu et al. 2014, 2018). This is further supported by our finding that an unexpected omission of aversive outcomes in a previous trial (i.e. indicating safety) was still followed by increased avoidance behaviour in the nicotine group, compared to placebo controls.

If we consider avoidance behaviour as another outcome measure that represents the response to aversive learning, our results may suggest that there is less discrimination between safety and danger in the nicotine group. Similarly, after acute nicotine before extinction (or before acquisition), participants showed reduced discrimination between threat and safety in fear retrieval (Mueller et al. 2024b). Chronic nicotine also reduced fear retrieval in mice and humans (Kutlu et al. 2018). Comparable results were found in another study where acute nicotine administration prior to extinction led to reduced freezing behaviour in mice compared to saline controls. Similar to the participants in our study, the mice showed altered, less safety-focused behaviour after nicotine administration during extinction (Elias et al. 2010). These findings could be explained by an impairment of memory retrieval and safety learning caused by nicotine, which is further supported by our results.

Another perspective is the exploration-exploitation dilemma, which describes the trade-off between the need for costly information gathering through exploration and strategic planning to use this knowledge to improve future behaviour (Berger-Tal et al. 2014). The perfect balance of exploration and exploitation during extinction would be to explore not to avoid the snakes and risk a potentially aversive outcome, but to learn that they are now safe and therefore exploit this knowledge to use the shorter path in the next trial. However, the nicotine group seemed to focus on exploration (choosing the path with more snakes), but ultimately failed to integrate this safety knowledge into their behaviour, compared to placebo controls.

This dilemma may also explain some of the findings in the paradigm itself. We found that participants took more steps in the lowest AOlevel of the medium path compared to the two more dangerous AOlevels. This could be a sign of reduced exploration in a context that is more difficult to judge than the short path level, where the cost of safety is certainly low, or the long path level, where the cost of safety is certainly high. In addition, there may be other mechanisms that explain behaviour in addition to the specifications provided by our paradigm. A recent study found thigmotactic behaviour, i.e. a tendency to stay closer to the walls in a potentially harmful environment, in participants exposed to a similar virtual context (Mueller et al. 2024a). Thigmotaxis may therefore also be a factor that is partly responsible for the behaviour that leads to an increased number of steps. Since we did not apply a comprehensive learning model to support the role of prediction errors in avoidance, there are alternative explanation for the effect observed in the nicotine group, e.g., a greater awareness for the overall change in contingencies that indicate that the context is safe.

To the best of our knowledge, previous evidence on the effects of acute nicotine on avoidance behaviour is very limited in animals and not yet described in humans. A previous study found that low doses of nicotine in mice reduced passive avoidance in a T-maze model, whereas higher doses increased passive avoidance (Nordberg and Bergh 1985). In line with this finding, nicotine administration led to improved learning of shuttle box avoidance in mice (Sansone et al. 1994) and increased active avoidance in a Sidman lever-press avoidance task in rats (Balfour and Morrison 1975).

In general, our new paradigm approach to measuring costly avoidance behaviour has been successful in showing differences between phases. As expected, we found the strongest avoidance in both groups during the acquisition phase. Then, during the uninstructed extinction phase, participants adapted to the change and showed less avoidance. Finally, during instructed extinction, avoidance behaviour decreased drastically. This was evident in both decision-making and effort-based analyses. In the task used here, participants faced an approach-avoidance conflict consisting of avoiding to-be-learned but fixed costs in the form of electric stimulation when approaching a fixed reward. Previous studies on decision-making in approach-avoidance conflicts have used tasks, in which monetary costs and rewards were both known a priori but varied in magnitude and probability (Korn and Bach 2019; Qi et al. 2018; Korn et al. 2017). Specifically, in some task variants a mobile predator probabilistically attacked participants on their path to collecting rewards. These studies found that the hippocampus, amygdala and prefrontal cortex play an integral role in computing protracted escape or avoidance decisions. The same neural network has been shown to be affected by nicotine administration during learning (Mueller et al. 2024b). Future research is needed to test if nicotine administration makes participants take more dangerous paths in such variants of approach-avoidance conflict tasks. Thereby, the underlying neural mechanisms of nicotine’s influence on avoidance could be clarified.

Our study has limitations. Each participant received the same dose of placebo or nicotine spray, independent of their weight. Further, the nicotine group reported stronger side effects, compared to placebo controls. However, these side effects were described as “very weak”. While our previous research and pilot studies suggest that 1 mg of nicotine provides a balance between efficacy and tolerability, future studies could explore different doses to further assess the robustness of nicotine’s effects on threat-related processing. It would also have been helpful to include plasma concentration measurements of nicotine levels for each participant. This may be a useful tool to investigate inter-individual differences in nicotine-induced effects. We did not collect data on race or ethnicity, but when looking at inter-individual differences it would also be very valuable to include this information in the analysis. Our exploratory decision-based model, including the binomial test, was not pre-registered, but was added because the path decision is a clearer representation of the choice made, compared to effort alone. Our experiment took about 30 min in total, during which time nicotine plasma levels may have changed slightly. However, nicotine has a half-life of two hours and the highest plasma nicotine levels in the brain are typically 20–30 min after peak (Benowitz et al. 1989; Feyerabend et al. 1985) Therefore, we would not expect a significant effect of changes in nicotine plasma levels between phases.

Overall, we can conclude that acute nicotine does influence the decision to engage in active avoidance behaviour in healthy non-smokers, but this is independent of effort. Nicotine administration, but not placebo, leads to a decrease in avoidance behaviour during uninstructed extinction. However, nicotine also leads to impaired safety learning during this phase, such that past experiences of strong safety are not as strongly implicated in avoidance behaviour as in placebo controls.

Nicotine is widely used, whether in the form of cigarettes or the increasingly popular e-cigarettes and highly addictive (Rigotti et al. 2022; Glasser et al. 2017). Moreover, studies have shown that patients with psychiatric disorders, such as anxiety, are more likely to smoke than the healthy population (Johnson et al. 2000; Cougle et al. 2010). Further, maladaptive avoidance behaviour is a key symptom of anxiety disorders (Salkovskis 1991; McManus et al. 2008). It is important to note that acute nicotine was administered to nicotine-naive participants in our study, who are likely to be more sensitive to nicotine exposure than chronic smokers. This is underlined by a previous study that found no effect of acute nicotine intake in smokers on fear learning or generalisation (Mueller et al. 2022). However, smoking in general has been found to impair safety learning in healthy individuals. Similar to the patterns seen in smokers, reduced threat-safety discrimination has also been demonstrated in nicotine-naive participants following a single dose of administered nicotine (Mueller et al. 2024b), similar to our pharmacological intervention. Future research on chronic nicotine use and its effects on avoidance behaviour and prediction error could help guide the use of nicotine based smoking cessation therapy. Examining how nicotine influences avoidance mechanisms could improve understanding of how extinction learning underlies changes in behaviour in the context of nicotine addiction. Furthermore, impaired safety learning and altered behaviour induced by nicotine use in healthy individuals may be a risk factor for the development of pathological anxiety that might fuel addictive behaviours and alter neurocircuits that process rewards (Koob and Volkow 2010).

## Electronic supplementary material

Below is the link to the electronic supplementary material.

Supplementary Material 1

## Data Availability

All data and code are available under this link: https://gin.g-node.org/MadeleineMueller/Mueller_et_al_2024_nicotine_and_avoidance.
