# Supplementary material for: The effect of nicotine on threat avoidance behaviour in healthy non-smokers
Source: Psychopharmacology (Berl). 2025 Apr 22;242(9):2111–21. doi: 10.1007/s00213-025-06789-9 (PMC12380871; doi:10.1007/s00213-025-06789-9)
Supplement: Supplementary file 1 — Supplementary Material 1 [file 213_2025_6789_MOESM1_ESM.docx]

Supplemental Material

-

The effect of nicotine on threat avoidance behaviour in healthy non-smokers

Madeleine Mueller, PhD ^1,2^, Christoph Korn, PhD ^1,3^ & Jan Haaker, PhD ^1^

^1^Department of Systems Neuroscience, University Medical Center Hamburg-Eppendorf, Hamburg, Germany

^2^Department of General Psychology and Cognitive Neuroscience, Charlotte Fresenius University, Hamburg, Germany

^3^Section Social Neuroscience, Department of General Psychiatry, University of Heidelberg, Heidelberg, Germany

METHODS


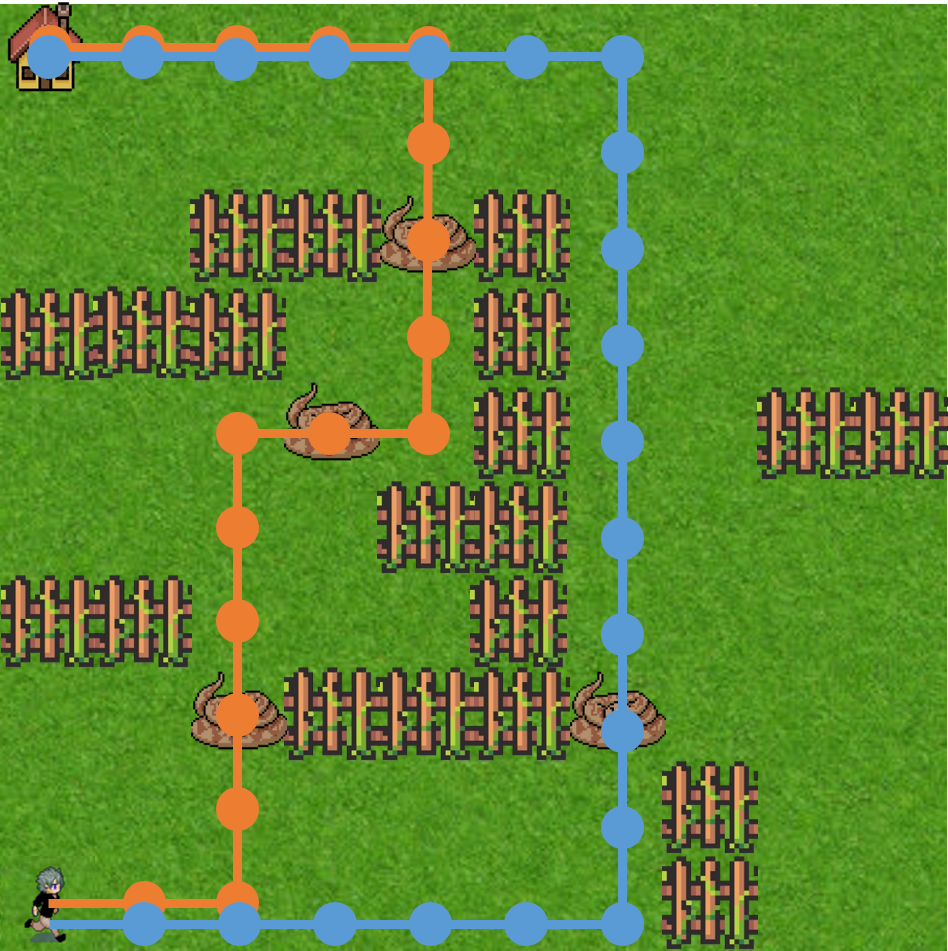


Figure S1: Example movement pattern when choosing either the short path (orange) or the longer path (blue). The shorter, but more dangerous path has a minimum of 17 steps, whereas the longer, but safer path has a minimum of 21 steps (in this version). Participants were free to move around, so that the actual number of steps could be higher than the minimum.

RESULTS

Decision-based effects + covariates

**Gender.** When gender was added as a covariate to our decision-based model (glm(pathdecision~AOlevel*Plevel*phase*group+gender), the follow-up ANOVA revealed a main effect of gender (F(1,2735)=22.35, p<0.001). Further post-hoc tests showed that women generally showed more avoidance behaviour, i.e. they chose the longer but safer path more often than men (female – male: z=4.80, p<0.001). The main effects reported in our main analysis were robust when gender was added as a covariate.

**Blinding success.** When blinding success was included in our decision-based model (glm(pathdecision~AOlevel*Plevel*phase*group+blinding_success), the follow-up ANOVA found no effect of this covariate (F(1,2915)=2.33, p=0.127). However, our results reported in the main analysis were robust to the addition of blinding success to the model.

Effort-based effects

Table S1: Effort-based effects. ANOVA results based on linear mixed model steps~(1|subject)+AOlevel*Plevel*phase*group. AOlevel = aversive outcome level; Plevel = pathlevel; ACQ = acquisition; EXT = extinction; fEXT = forced extinction.

|  | F | Df | p |
| --- | --- | --- | --- |
| AOlevel | 1.44 | 2,2852 | 0.237 |
| **Plevel** | **50.29** | **2,2852** | **<0.001** |
| **Phase** | **11.37** | **2,2852** | **<0.001** |
| group | 0.01 | 1,240.66 | 0.922 |
| **AOlevel*Plevel** | **2.74** | **4,2852** | **0.027** |
| AOlevel*phase | 0.57 | 4,2852 | 0.681 |
| **Plevel*phase** | **4.26** | **4,2852** | **0.002** |
| AOlevel*group | 0.05 | 2,2852 | 0.947 |
| Plevel*group | 0.31 | 2,2852 | 0.735 |
| Phase*group | 1.44 | 2,2852 | 0.236 |
| AOlevel*Plevel*phase | 1.51 | 8,2852 | 0.149 |
| AOlevel*Plevel*group | 0.92 | 4,2852 | 0.452 |
| AOlevel*phase*group | 0.29 | 4,2852 | 0.886 |
| Plevel*phase*group | 0.72 | 4,2852 | 0.579 |
| AOlevel*Plevel*phase*group | 1.11 | 8,2852 | 0.353 |

Table S2: Effort-based effects. Only results of post-hoc tests (estimated marginal means; Bonferroni-Holm corrected) based on significant effects found in the ANOVA described in Table S1 are shown. AOlevel = aversive outcome level; Plevel = pathlevel; ACQ = acquisition; EXT = extinction; fEXT = forced extinction.

|  | t | df | p_corr_ |
| --- | --- | --- | --- |
| **Plevel** |  |  |  |
| Plevel1 – Plevel2 | -18.17 | 2852 | <0.001 |
| Plevel1 – Plevel3 | -30.67 | 2852 | <0.001 |
| Plevel2 – Plevel3 | -12.5 | 2852 | <0.001 |
| **Phase** |  |  |  |
| ACQ – EXT | 7.66 | 2852 | <0.001 |
| ACQ – fEXT | 34.16 | 2852 | <0.001 |
| EXT – fEXT | 27.91 | 2852 | <0.001 |
| **AOlevel*Plevel (within Plevel)** |  |  |  |
| lowest Plevel/lowest AOlevel - lowest Plevel/medium AOlevel | 1.44 | 2852 | 0.902 |
| lowest Plevel/lowest AOlevel - lowest Plevel/highest AOlevel | -0.25 | 2852 | 0.802 |
| lowest Plevel/medium AOlevel - lowest Plevel/highest AOlevel | -1.69 | 2852 | 0.638 |
| medium Plevel/lowest AOlevel - medium Plevel/medium AOlevel | 3.84 | 2852 | <0.001 |
| medium Plevel/lowest AOlevel - medium Plevel/highest AOlevel | 3.07 | 2852 | 0.018 |
| medium Plevel/medium AOlevel - medium Plevel/highest AOlevel | -0.77 | 2852 | 1 |
| highest Plevel/lowest AOlevel - highest Plevel/medium AOlevel | -0.37 | 2852 | 1 |
| highest Plevel/lowest AOlevel - highest Plevel/highest AOlevel | -0.87 | 2852 | 1 |
| highest Plevel/medium AOlevel - highest Plevel/highest AOlevel | -0.50 | 2852 | 1 |
| **AOlevel*Plevel (within AOlevel)** |  |  |  |
| lowest AOlevel/lowest Plevel – lowest AOlevel/medium Plevel | -12.40 | 2852 | <0.001 |
| lowest AOlevel/lowest Plevel – lowest AOlevel/highest Plevel | -16.90 | 2852 | <0.001 |
| lowest AOlevel/medium Plevel – lowest AOlevel/highest Plevel | -4.50 | 2852 | <0.001 |
| medium AOlevel/lowest Plevel – medium AOlevel/medium Plevel | -9.996 | 2852 | <0.001 |
| medium AOlevel/lowest Plevel – medium AOlevel/highest Plevel | -18.71 | 2852 | <0.001 |
| medium AOlevel/medium Plevel – medium AOlevel/highest Plevel | -8.71 | 2852 | <0.001 |
| highest AOlevel/lowest Plevel – highest AOlevel/medium Plevel | -9.08 | 2852 | <0.001 |
| highest AOlevel/lowest Plevel – highest AOlevel/highest Plevel | -17.52 | 2852 | <0.001 |
| highest AOlevel/medium Plevel – highest AOlevel/highest Plevel | -8.44 | 2852 | <0.001 |
| **Plevel*phase (within Plevel)** |  |  |  |
| lowest Plevel/ACQ – lowest Plevel/EXT | 1.996 | 2852 | 0.092 |
| lowest Plevel/ACQ – lowest Plevel/fEXT | 9.95 | 2852 | <0.001 |
| lowest Plevel/EXT – lowest Plevel/fEXT | 8.32 | 2852 | <0.001 |
| medium Plevel/ACQ – medium Plevel/EXT | 5.04 | 2852 | <0.001 |
| medium Plevel/ACQ – medium Plevel/fEXT | 19.59 | 2852 | <0.001 |
| medium Plevel/EXT – medium Plevel/fEXT | 15.47 | 2852 | <0.001 |
| highest Plevel/ACQ – highest Plevel/EXT | 6.22 | 2852 | <0.001 |
| highest Plevel/ACQ – highest Plevel/fEXT | 29.62 | 2852 | <0.001 |
| highest Plevel/EXT – highest Plevel/fEXT | 24.54 | 2852 | <0.001 |
| **Plevel*phase (within phase)** |  |  |  |
| ACQ/lowest Plevel – ACQ/medium Plevel | -17.06 | 2852 | <0.001 |
| ACQ/lowest Plevel – ACQ/highest Plevel | -29.89 | 2852 | <0.001 |
| ACQ/medium Plevel – ACQ/highest Plevel | -12.83 | 2852 | <0.001 |
| EXT/lowest Plevel – EXT /medium Plevel | -14.02 | 2852 | <0.001 |
| EXT /lowest Plevel – EXT /highest Plevel | -25.66 | 2852 | <0.001 |
| EXT /medium Plevel – EXT /highest Plevel | -11.64 | 2852 | <0.001 |
| fEXT/lowest Plevel – fEXT /medium Plevel | -3.72 | 2852 | <0.001 |
| fEXT /lowest Plevel – fEXT /highest Plevel | -4.10 | 2852 | <0.001 |
| fEXT/medium Plevel – fEXT /highest Plevel | -0.38 | 2852 | 0.705 |
